# Effects of Iron and Zinc on Mitochondria: Potential Mechanisms of Glaucomatous Injury

**DOI:** 10.3389/fcell.2021.720288

**Published:** 2021-08-10

**Authors:** Jiahui Tang, Yehong Zhuo, Yiqing Li

**Affiliations:** State Key Laboratory of Ophthalmology, Zhongshan Ophthalmic Center, Sun Yat-sen University, Guangzhou, China

**Keywords:** glaucoma, retinal ganglion cell, mitochondria, mitophagy, iron, zinc, ferroptosis

## Abstract

Glaucoma is the most substantial cause of irreversible blinding, which is accompanied by progressive retinal ganglion cell damage. Retinal ganglion cells are energy-intensive neurons that connect the brain and retina, and depend on mitochondrial homeostasis to transduce visual information through the brain. As cofactors that regulate many metabolic signals, iron and zinc have attracted increasing attention in studies on neurons and neurodegenerative diseases. Here, we summarize the research connecting iron, zinc, neuronal mitochondria, and glaucomatous injury, with the aim of updating and expanding the current view of how retinal ganglion cells degenerate in glaucoma, which can reveal novel potential targets for neuroprotection.

## Introduction

Glaucoma involves irreversible optic nerve injury, which remains an urgent clinical challenge affecting 3–4% of people over 40 years of age; the global prevalence of glaucoma is expected to escalate to approximately 112 million people by 2040 (Tham et al., 2014). Patients with glaucoma often experience vision loss, which negatively impacts their independence and quality of life (Fenwick et al., 2020). One hallmark of glaucoma is progressive damage to retinal ganglion cells (RGCs) (Gupta and Yucel, 2007). However, the mechanisms of RGC degeneration in glaucoma are not yet completely understood. Medical and surgical intraocular pressure (IOP) control is the most common treatment for glaucoma at present (Kass et al., 2002; Osborne, 2008). However, these traditional therapies can neither maintain the long-term survival of RGCs nor promote optic nerve regeneration, strongly suggesting the presence of unknown molecular mechanisms (Osborne, 2011).

Recent evidence points to mitochondrial dysfunction in the retina, especially in RGCs, as an emerging hypothesis for glaucoma pathogenesis, offering a potential novel target for intervention. In the retinal structure, the somata of RGCs are located in the ganglion cell layer, with dendrites projecting to the inner plexiform layer. The long axons extend through the optic nerve. Through synapses, the dendrites and axons connect with other retinal neurons and cellular partners in the brain, respectively. These synapses require large amounts of energy for neurotransmitter synthesis, synaptic vesicle assembly, ion gradient formation, and calcium buffering (Vos et al., 2010). Mitochondria in RGCs play an essential role in meeting this high energy demand (Chidlow et al., 2019). Mitochondria play a pivotal role in metabolism and cell death (e.g., tricarboxylic acid cycle, ATP production, and apoptosis), and dysfunctional mitochondria can result in various diseases (Nicholls and Budd, 2000; Devine and Kittler, 2018; Pfanner et al., 2019). Mitochondria maintain homeostasis and quality control through fission, fusion, mitophagy, and biogenesis (Youle and van der Bliek, 2012; Dorn, 2019). Although studies have unveiled the many critical molecules and pathways related to the roles of mitochondria in neurons, there are many unsolved questions regarding the existence and functions of these molecules and pathways in RGCs.

Metal ions such as iron and zinc are essential for normal cellular function, especially in the synapses of the nervous system, and both ions are critical cofactors of neurotransmitter synthesis (Rouault, 2013). In particular, zinc modulates synaptic activities (Sourkes, 1972; McAllister and Dyck, 2017) and acts as an intracellular second messenger (Yamasaki et al., 2007). In addition to the free form ion, metal ion signals are transduced by metalloproteins to affect specific intracellular functions. The crucial participation of these metal ions in the pathogenesis and progression of neurodegenerative diseases such as Alzheimer’s disease (Liu et al., 2019) and Parkinson’s disease (Mocchegiani et al., 2005; Derry et al., 2020), has attracted increasing interest. Although only a few studies have directly focused on the roles of metal ions in glaucoma, we can infer from the existing literature that dysregulation of mitochondrial metal ions is the primary pathogenic cause of glaucomatous injury, rendering these ions as the most promising targets for therapy. In this review, we briefly describe the coordination among mitochondria, iron, and zinc; elucidate the mechanism underlying the mitochondrial homeostasis of RGCs in glaucoma; and discuss the emerging roles of iron and zinc in the mitochondria-related pathogenesis of glaucomatous RGCs.

## Metal Ions and Mitochondria

**FIGURE 1:**
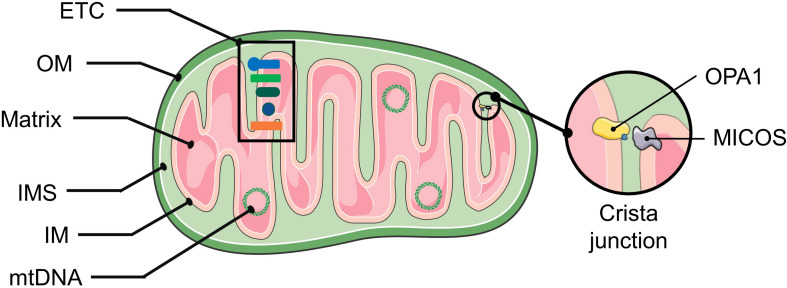
The structure of mitochondria. The mitochondrial double membrane is a tubular reticulum, including an outer membrane (OM) and inner membrane (IM). The OM is porous and freely permits the diffusion of molecules smaller than 5 kD. The IM is a barrier to free ion diffusion and contains several cations and metabolite transporters. The invaginations of the IM that increase the surface area are called cristae. The inner mitochondrial membrane encloses the mitochondrial matrix. An intermembrane space (IMS) exists between the OM and IM, and its composition is close to that of the cytoplasmic matrix. The crista junction represents the architecture by which the cristae are attached to the inner membrane via narrow stems (Panek et al., 2020). The MICOS complex and OPA1 are involved in shaping the cristae. OPA1 act as a tether that maintains “tight” junctions to sequester cytochrome c and oxidative-phosphorylation protein complexes within the crista membrane (Mannella, 2020). Abbreviations: ETC, electron transport chain; mtDNA, mitochondrial DNA; MICOS, mitochondrial contact site and cristae organization system.

More than 1000 biochemical reactions responsible for cellular functions occur in mitochondria, which extend far beyond their well-known function of ATP synthesis. These vital functions depend on the structural composition of mitochondria (Figure 1). Mitochondria are incredibly dynamic double-membrane-bound subcellular organelles, comprising more than 1200 proteins, for which the composition varies substantially in different tissues or at different developmental stages. Many of these proteins are metalloproteins (Table 1).

**TABLE 1:** Mitochondrial metalloproteins and associated functions.

Category	Proteins	Localization	Regulatory functions	References
Mitochondrial iron metalloproteins	Isu1 Isu2	Matrix	*De novo* synthesis of Fe-S clusters	Muhlenhoff et al., 2003; Braymer and Lill, 2017
	Ferredoxin	Matrix	Synthesis of Fe-S clusters	Smith et al., 1991; Lange et al., 2000
			Mediates electron transfer	
	Aconitase	Matrix/Cytoplasm	Part of the citric acid cycle	Mascotti et al., 1995; Rouault, 2006
			Controls iron homeostasis	
	FtMt	Matrix	Controls iron homeostasis	Levi et al., 2001
	Frataxin	Matrix	Synthesis of Fe-S clusters	Bulteau et al., 2004
Mitochondrial zinc	AFG3L2	Matrix/IM	Ribosome assembly	Kondadi et al., 2014; Konig et al., 2016
metalloproteins	SPG7		MCU assembly	
	YME1L	IM/IMS	Protein import	Potting et al., 2013;
			Lipid trafficking	MacVicar and Langer, 2016;
			Mitochondrial dynamics	Richter et al., 2019
	OMA1	IMS/IM	Mitochondrial dynamics	Anand et al., 2014
	MPP	Matrix	Maturation of mitochondrial proteins	Mossmann et al., 2012
	MIP	Matrix	Protein maturation	Branda and Isaya, 1995;
			Coenzyme Q biosynthesis	Allan et al., 2015
	Atp23	IMS	Protein maturation	Osman et al., 2007; Zeng et al., 2007
			F_1_F_O_-ATP synthase assembly	

*Abbreviations: IM, inner membrane; IMS, intermembrane space; MCU, mitochondrial Ca^2+^ uniporter.*

### Mitochondrial Iron Metalloproteins

Iron is one of the most abundant metals in mitochondria, which is stored in mitochondrial ferritin (FtMt) within the mitochondrial matrix (Levi et al., 2001). Mitochondria require iron for the respiratory chain polynuclear sulfur-bridged iron-sulfur (Fe/S) centers residing in the cristae membrane. Fe/S clusters exist in respiratory complexes I, II, and III, and are critical for oxidative phosphorylation, which is the process by which electrons from NADH and FADH_2_ are transferred to O_2_ molecules through a series of electron carriers/protein complexes. This process generates potential energy in the form of a pH gradient and an electrical potential across the membrane to synthesize ATP from ADP to meet the cell’s energetic needs (Harper et al., 2018). Fe/S centers of different nuclearities are present in numerous proteins, in addition to those associated with the iron-sulfur cluster machinery within the mitochondrial matrix, such as the Fe/S scaffold proteins Isu1 and Isu2 (Andrew et al., 2008). These proteins include ferredoxin, biotin synthase, and aconitase (Lill and Muhlenhoff, 2008).

### Mitochondrial Zinc Metalloproteins

Many zinc metalloproteins function in the mitochondria, including iAAA, mAAA, OMA1, mitochondrial processing peptidase (MPP), mitochondrial intermediate peptidase (MIP), and Atp23. Mitochondrial proteins are encoded by both nuclear DNA and mitochondrial DNA (mtDNA), which must be transported into the mitochondria for correct folding. Because mitochondrial electron transport chains can generate toxic reactive oxygen species (ROS), mitochondrial proteins are easily damaged. These processes make proteases particularly important. Mitoproteases are divided into four functional categories as follows: ATP-dependent peptidases, processing peptidases, oligopeptidases, and other mitochondrial peptidases (Deshwal et al., 2020).

ATP-dependent proteases, including iAAA and mAAA, perform quality control and regulatory functions in the mitoprotease system. The iAAA protease consists of six mobile zinc (Zn^2+^)-binding yeast mtDNA escape 1-like (YME1L) subunits and is active at the intermembrane space (IMS) side (Leonhard et al., 2000; Puchades et al., 2017). iAAA regulates mitochondrial protein degradation, lipid trafficking, and mitochondrial dynamics (Shi et al., 2016; MacVicar et al., 2019; Sprenger et al., 2019). The mAAA protease is composed of AFG3-like subunit 2 (AFG3L2) or spastic paraplegia 7-homolog (SPG7) subunits (Yta10/Afg3l2 and Yta12/Rca1 in yeast, respectively, each requiring Zn^2+^ for activity) and is active on the matrix side (Koppen et al., 2007). mAAA might function together with iAAA and OMA1 to regulate optic atrophy 1 (OPA1), a vital factor in mitochondrial dynamics (Consolato et al., 2018).

MPP cleaves mitochondrial-targeting sequences in the matrix for the maturation of nucleus-encoded mitochondrial proteins. The mitochondrial intermediate peptidase MIP (Oct1 in yeast) cleaves off the N-terminal octapeptide of some proteins for their stabilization (Vogtle et al., 2011). Atp23 forms an inner membrane protease to mediate the maturation of some proteins such as F_1_F_0_-ATP synthase into the IMS (Osman et al., 2007). However, protein processing removes the targeting sequences and acts as a regulatory mechanism that determines the activity and localization of mitochondrial proteins. As mentioned above, the inner membrane metalloendopeptidase OMA1 has some joint functions with mAAA to mediate proteolytic processes (Rainbolt et al., 2016; Consolato et al., 2018). The relationship between zinc-dependent proteases and mitochondrial dynamics has received increasing attention (further discussion in section “Effects of Zinc on Mitochondria”).

## Movement of Metal Ions

As mentioned above, metalloproteins are distributed and function throughout the mitochondria. Therefore, in this section, we summarize the acquisition, distribution, transportation, storage, and exportation of metal ions in a cell and mitochondria.

### Cellular and Mitochondrial Iron

As for the acquirement of cellular iron. The extracellular ferric form of iron combined with transferrin binds to transferrin receptor 1 and enters the cell via endocytosis (Picard et al., 2020). Transferrin receptors in neurons are expressed in the soma and dendrites but not in the axon (West et al., 1997). Upon maturation and acidification, endosomes release iron, which is reduced to the ferrous form. Divalent metal transporter-1 (DMT1, also known as NRAMP2, DCT1, or SLC11A2), transient receptor potential mucolipin 1 (TRPML1), and ZRT/IRT-like protein (ZIP14) mediate iron transport from the endosome to the cytoplasm (Upadhyay and Agarwal, 2020). Extracellular non-transferrin-bound Fe^2+^ can be directly internalized to the labile iron pool (LIP) by DMT1 on the cell surface (Moos et al., 2007). As for the storage of cellular iron. Ferritin, the principal iron-storage protein, is comprised of heavy (H)- and light (L)-chain monomers. The H-chain subunit oxidizes Fe^2+^ to Fe^3+^ via its ferroxidase activity to enhance iron sequestration by ferritin (Muhoberac and Vidal, 2013), whereas the L-subunit stores more iron by facilitating iron core formation (Ashraf et al., 2018). Fe^2+^ in the cytoplasm transiently enters the LIP. Notably, the ferrous form can cause molecular and cellular dysfunction by catalyzing the formation of hydroxyl free radicals (⋅OH) via the Fenton reaction (Ueda et al., 2018). However, the studies focused on cellular iron efflux are limited. Ferroportin (Fpn) is the only known cellular iron exporter that requires Ca^2+^ as a cofactor (Deshpande et al., 2018). Collectively, these studies suggest that RGCs take up iron at the cell body and dendrites, and ferritin and Fpn combine to export iron both within the axon and elsewhere.

Mitochondria acquire iron directly from both endosomes and the cytosol. Iron utilizes voltage-dependent anion channels and DMT1 to cross the outer membrane (Szabo and Zoratti, 2014; Wolff et al., 2018). The transport of Fe^2+^ across the inner membrane and its import into the matrix requires the mitoferrins Mfrn1 and Mfrn2 (orthologs in yeast are Mrs3/4), also known as SLC25A37 and SLC25A28, respectively (Grillo et al., 2017). Mitoferrin deficiency impairs iron import into the mitochondrial matrix via disruption of Mfrn1 and Mfrn2 in mammalian cells and of Mrs3 and Mrs4 in yeast, resulting in impaired iron metabolism and mitochondrial [Fe-S] cluster biogenesis (Chung et al., 2014). FtMt is an iron-storage protein that specifically functions in the mitochondria and cooperates with cytosolic ferritin to regulate iron homeostasis in both the cytoplasm and mitochondria (Drysdale et al., 2002). FtMt can suppress Fe^2+^-induced mitochondrial ROS production (Yang et al., 2013). Moreover, FtMt overexpression has been shown to ameliorate several neurodegenerative diseases (Gao and Chang, 2014). The neuroprotection mechanism of FtMt is considered to involve inhibiting the elevation of LIP levels and ferroptosis, as a new type of iron-dependent regulated cell death (Wang et al., 2016). However, there are few reports on the channels responsible for iron export from the mitochondria. Overexpression of Mmt1/2 in yeast leads to a low-iron transcriptional response, which can also be seen in Mrs3- and Mrs4-knockout cells. Therefore, Li et al. (2014) hypothesized that Mmt1/2 functions as a mitochondrial iron exporter. Mmt1 and Mmt2 expression is transcriptionally regulated by the low iron–sensing transcription factor Aft1 and the oxidant-sensing transcription factor Yap1 to accommodate changes in cytoplasmic and mitochondrial iron (Tuncay et al., 2019).

### Cellular and Mitochondrial Zinc

Zinc transporters SLC39s/ZIPs (ZRT/IRT-like proteins) increase cytoplasmic zinc concentrations by translocating Zn^2+^ from the extracellular space or organelles into the cytoplasm (Eide, 2006; Baltaci and Yuce, 2018). In addition to ZIPs, the uptake of extracellular Zn^2+^ also occurs through amino-3-hydroxy-5-methyl-4-isoxazolepropionic acid receptors (AMPARs) (Yin and Weiss, 1995; Sensi et al., 1999), voltage-gated calcium channels (VGCCs) (Kerchner et al., 2000), and N-methyl-D-aspartate receptors (NMDARs) (Koh and Choi, 1994). The translocated Zn^2+^ then activates numerous physiological and pathophysiological signaling processes. The majority (∼90%) of cellular zinc in neurons is found in a tightly protein-bound form (Huang, 1997). Under various injurious stimuli, zinc liberation from cytosolic zinc metalloproteins can lead to an increase in the intracellular Zn^2+^ level. The accumulated Zn^2+^ has three potential fates: (1) transport into the mitochondria and other subcellular compartments such as the endoplasmic reticulum; (2) formation of a labile zinc pool in the cytosol; and (3) enter the synaptic vesicles, followed by its transportation out of the cell to post-synaptic neurons. As for zinc export, SLC30s/ZnTs (zinc transporters) reduce the cytoplasmic zinc concentration by translocating Zn^2+^ from the cytoplasm into the extracellular space or organelles (Jackson et al., 2007). ZnT-3 is responsible for loading Zn^2+^ into the synaptic vesicles of glutamatergic, monoaminergic, and GABAergic neurons (Palmiter et al., 1996). In the retina, ZnT-3 is also a key regulator of Zn^2+^ transport to the synaptic vesicles of amacrine cells and melanopsin-containing RGCs (Li et al., 2017; Moshirpour et al., 2020).

The zinc ion transport channel in neural mitochondria has not yet been identified. Previous studies have shown that Zn^2+^ enters the mitochondria through the mitochondrial calcium uniporter (MCU) to trigger mitochondrial dysfunction (Gazaryan et al., 2007; Ji et al., 2019). A South Korean research team found that ZIP1, located in the mitochondrial outer membrane, and MCU, located in the mitochondrial inner membrane, work together to mediate zinc ion entry to the mitochondrial matrix after dynamin-1-like protein (DRP1) is activated in primary rat cortical neurons (Cho et al., 2019). This mechanism links zinc with the mitochondrial membrane potential and mitochondrial division, which provides new insight into the role of mitophagy in neurodegenerative diseases (Cho et al., 2019; Ji et al., 2020). ZIP1 is widely expressed in the central nervous system, and is localized in both the cytoplasmic membrane and cytoplasm (Tang et al., 2006; Qian et al., 2011). Tuncay et al. (2019) provided an essential description of the roles of ZIP7 and ZnT7, demonstrating that the proteins are localized in both the mitochondria and the sarco(endo)plasmic reticulum, and contribute to cellular Zn^2+^ exchange between subcellular compartments in cardiomyocytes under hyperglycemia or hypertrophy by affecting sarco(endo)plasmic reticulum-mitochondria coupling (Tuncay et al., 2019). Increasing ZnT7 and decreasing ZIP7 levels in mitochondria induces higher mitochondrial free Zn^2+^ levels, ROS production, and a depolarized mitochondrial membrane potential (Tuncay et al., 2019). However, the existence of these mechanisms in neurons or RGCs should be confirmed in further studies.

## Metal Homeostasis and the Fate of RGCs

Retinal and humoral metal levels have been assessed in the context of glaucoma and other neurodegenerative diseases. Inductively coupled plasma-mass spectrometry analysis showed that retina and optic nerve samples from the DBA/2J glaucoma mouse model had lower iron concentrations than those of the retina from age-matched C57BL/6J control mice. Moreover, the retina of pre-glaucomatous DBA/2J mice showed over twofold higher zinc concentrations than those in 10-month-old DBA/2J retina (DeToma et al., 2014). Nevertheless, consuming iron above a threshold can increase the risk of developing glaucoma (Wang et al., 2012). Synchrotron X-ray fluorescence of the choroidal stroma in aged old-world primates confirmed the focal accumulation of iron (Ugarte et al., 2018). Compared with the control retina, glaucomatous retina of monkeys and humans show increased mRNA expression levels of iron-regulating genes (Farkas et al., 2004). Similarly, high serum ferritin levels were reported to be independently associated with a greater risk for human glaucoma (Gye et al., 2016).

With a greater understanding of the relationships among glaucoma, iron, and zinc, it is important to further elucidate the mechanism of metal dyshomeostasis in RGCs and the subsequent cellular pathophysiological signaling processes.

### Iron Homeostasis and the Fate of RGCs

The iron steady state regulated by mitochondrial iron-binding proteins is essential for RGC survival. Frataxin (FXN) is a highly conserved nuclear-encoded mitochondrial protein among metabolically active eukaryotes (Adinolfi et al., 2009). FXN is an iron chaperone protein that protects against aconitase [4Fe-4S]^2+^ disassembly and promotes enzyme reactivation, preserving mitochondrial iron homeostasis and Kreb cycle functions. FXN stores iron within the mitochondria and promotes Fe^2+^ availability. Aconitase is highly susceptible to oxidation and inactivation following the release of solvent-exposed Fe-α and the formation of a [3Fe-4S]^1+^ cluster (Bulteau et al., 2004). Friedreich’s ataxia is a neurodegenerative and cardiac disorder characterized by FXN deficiency, resulting in mitochondrial iron accumulation and impaired activity of aconitase along with other mitochondrial iron-sulfur proteins (Ast et al., 2019). The transient elevation of IOP causes retinal endogenous FXN upregulation (Schultz et al., 2016). Whole-body or Müller cells overexpressing FXN protect RGCs after acute ischemia/reperfusion (I/R) injury (Schultz et al., 2016, 2018). Improved RGC survival is associated with increased antioxidative responsivity and mitochondrial functional maintenance.

Iron homeostasis regulated by neurotransmission is essential for RGC survival. Glutamate-mediated neurotransmission translates visual information from photoreceptors to bipolar cells, RGCs, and brain centers (Lukasiewicz, 2005). Excess glutamate in the retina underlies common neurodegenerative disorders such as retinal artery occlusion and glaucoma (Pang and Clark, 2020). The mechanism of excitotoxic injury involves the binding of excess glutamate to cell-surface NMDARs, resulting in a toxic influx of calcium, ultimately leading to RGC death (Almasieh et al., 2012). Moreover, glutamate-induced NMDAR activation was found to increase iron uptake through the iron import channel DMT1 (Cheah et al., 2006; Chen Y. et al., 2013). A more recent study demonstrated that intravitreal NMDA causes iron accumulation in RGCs and triggers apoptosis in these neurons (Sakamoto et al., 2018). Iron-chelating agents such as deferoxamine (DFO) and deferasirox (DFX) protect RGCs against excitoneurotoxicity or IOP perturbations by reducing the intracellular iron content and oxidative stress in rats (Liu et al., 2014; Sakamoto et al., 2018).

In addition to excitotoxicity, oxidative stress leads to RGC death in experimental models of optic nerve injury and in human glaucoma. The intracellular ROS triggered by axonal injury has also been proposed to be a key death signal leading to RGC apoptosis (Almasieh et al., 2012). As discussed earlier, intracellular ferrous iron catalyzes the conversion of hydrogen peroxide to hydroxyl radicals (⋅OH). Fe^2+^ also catalyzes the oxidation of lipid peroxide to lipid alkoxyl radicals via the Fenton reaction (Pollitt, 1999) as a key process of ferroptosis. However, under oxidative stress, superoxide also induces Fe^2+^ release from iron metalloproteins, including iron-sulfur clusters and ferritin (Aliaga et al., 2011). The degradation of ferritin via ferritinophagy is mediated by nuclear receptor coactivator 4, which increases the cytosolic LIP to ultimately enhance ferroptosis (Mancias et al., 2014). Thus, the cellular redox state and Fe^2+^ availability mutually interact to develop a positive feedback loop in RGCs.

**FIGURE 2:**
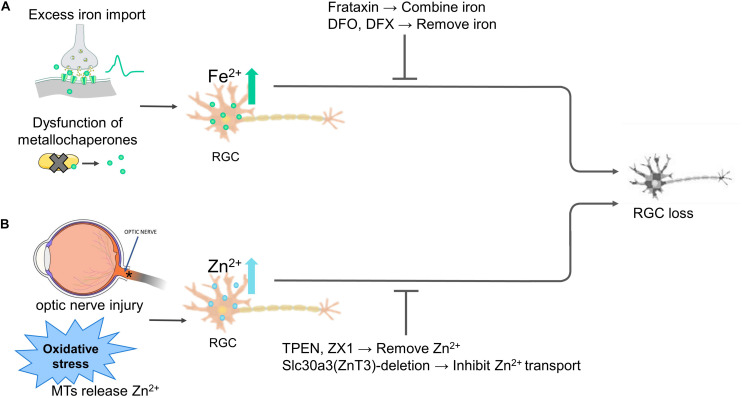
Iron and zinc homeostasis regulate the fate of retinal ganglion cells (RGCs). **(A)** Overly extracellular iron import, iron-binding proteins dysfunction disturbs intracellular iron levels, both causes RGC death. Overexpressing frataxin or the use of iron-chelating agents deferoxamine (DFO) and deferasirox (DFX) to stabilize or remove the excessive Fe^2+^ alleviates RGC death. **(B)** Optic nerve injury and various stimuli such as oxidative stress liberate Zn^2+^ from MTs, inhibiting RGC survival and axon regeneration. Zinc transporter Slc30a3-deletion or the Zn^2+^ chelator TPEN and ZX1 treatment inhibit transport or chelate Zn^2+^, protecting RGC survival and axon regeneration. Abbreviations: MTs, metallothioneins.

These studies indicate that metallochaperones such as FXN, iron transporters such as DMT1, and the cellular redox state collectively regulate iron homeostasis. Thus, dysfunction in a metallochaperone, activation of iron import channels, and/or oxidative stress will result in iron dyshomeostasis and subsequent death of RGCs (Figure 2A).

### Zinc Homeostasis and the Fate of RGCs

Intracellular zinc is stored within the retina in RGCs, horizontal cells, and amacrine cells (Akagi et al., 2001; Kaneda et al., 2005). Most of the intracellular zinc is tightly bound, compartmentalized, and sequestered with high affinity by proteins, including metallothioneins (MTs), facilitating specific processes. As a consequence of these high-affinity binding events, the concentration of available zinc, referred to as “free,” “labile,” “mobile,” or “exchangeable” zinc, for metabolic processes is tightly regulated (Gilbert et al., 2019). Upon perturbation, cytosolic zinc metalloproteins respond to transient cytosolic zinc ion concentration changes, which is termed “zinc muffling” (Tuncay et al., 2019).

Excess intracellular zinc promotes the loss of RGCs. The Zn^2+^ levels of RGCs can vary depending on many factors, including Zn^2+^ release from subcellular zinc stores and zinc ion influx via channel activity. Under oxidative stress conditions, ROS and peroxynitrite oxidize residues on the metal-binding sites of metal-binding proteins and release the mobile zinc (Hidalgo et al., 2001). Peroxynitrite is an oxidant produced by the reaction between nitric oxide (NO) and superoxide radicals. Interestingly, the production of NO after optic nerve injury might act upstream of zinc liberation, leading to accumulation in amacrine cell terminals (Sergeeva et al., 2021). The accumulation of Zn^2+^ in the synaptic contacts between the amacrine cells and dendrites of RGCs is one of the earliest events following optic nerve injury (Benowitz et al., 2017). Li et al. (2017) observed that retinal amacrine cell Zn^2+^ concentrations increase by several-fold following optic nerve injury, and the excess is secreted in vesicles to RGCs. Zn^2+^ accumulation in RGCs is a newly discovered cause of axonal degeneration and apoptosis. However, zinc chelation reduces intracellular zinc concentrations, promoting RGC survival and axon regeneration (Li et al., 2017). These results show that excess intracellular zinc promotes the loss of RGCs.

The high levels of cellular zinc make RGCs susceptible to excitotoxic apoptosis. In addition to NMDARs, GABA/glycine receptors exist in retinal ganglion cells, which inhibit neurotransmission and protect RGCs against excitotoxic episodes (Hadj-Said et al., 2017). At concentrations below 10 μM (Kaneda et al., 2005), Zn^2+^ binds to the high-affinity site of glycine receptors, which facilitates glycine binding. As the concentration increases to 50 μM or more (Kaneda et al., 2005), Zn^2+^ binds to the low-affinity site. This binding inhibits Zn^2+^ binding to high-affinity sites on glycine receptors. The low-affinity site is located near the glycine-binding pocket, acting as a competitive inhibitor of glycine receptors (Han and Wu, 1999).

These studies indicate that oxidative stress, optic nerve injury, dysfunction of zinc-binding proteins result in zinc dysregulation and subsequent susceptibility to excitotoxic and death of RGCs (Figure 2B).

## Mitochondria of RGCs: Critical Target of Metal Ions

The mitochondria are highly interconnected, and their synchronous intracellular functions make them unique among organelles. Mitochondria are continually recycled in a dynamic balance termed mitochondrial homeostasis, including mitochondrial genesis, movement, fission, fusion, and mitophagy.

### Mitochondrial Biogenesis, Motility, Dynamics, and Mitophagy

Mitochondria produce ∼1300 proteins from the nuclear genome, and 13 proteins are solely encoded by vertebrate mitochondrial DNA, reflecting the truly symbiotic nature of this organelle (Schapira, 2012; Alston et al., 2021). Mitochondrial biogenesis mainly occurs near the nucleus to ensure the incorporation of only correctly synthesized molecules. Due to their bacterial nature, new mitochondria result from the fission of older mitochondria. Hence, some mitochondria remain in the neuronal soma to generate new mitochondria.

Other mitochondria must be trafficked to axons and dendrites, and positioned carefully to meet the demand of energy-consuming sites such as the pre-synaptic terminals. However, as mitochondrial transport can be accomplished in ∼0.5 μm/s and many nuclear-encoded mitochondrial proteins have short half-lives, we speculate that non-canonical local mitogenesis must also take place within neurons (Kaplan et al., 2009; Price et al., 2010), and that both mitogenic events are regulated by peroxisome proliferator-activated γ coactivator 1α (PGC-1α) (Fernandez-Marcos and Auwerx, 2011). However, the exact relationship between mitochondrial soma biogenesis and local biogenesis is not yet clear.

Because of the heterogeneous distribution and synthesis of mitochondria, in RGCs, one group of mitochondria undergoes anterograde and retrograde transport under control of the kinesin-1 motor protein family (Kif5B in mammals) and dynein proteins, respectively (Shanmughapriya et al., 2020). The membrane-anchored Miro (also called RhoT1/2) and its motor-binding partner Milton (also called TRAK1/2) form a Miro-Milton complex that cooperates with the kinesin-1 motor and dynein to mediate anterograde and retrograde transport (Schwarz, 2013). Another group of mitochondria remains anchored to the microtubules via syntaphilin, a mitochondrial outer membrane-attached protein, and an actin cytoskeleton component (Pathak et al., 2010). Interestingly, syntaphilin is only expressed in the soma and not in the axon of rat RGCs, suggesting that axons regulate their stationary pool of mitochondria differently from other neurons (Miki et al., 2014). Mitochondrial trafficking can be visualized via video microscopy using a fluorescent dye such as tetramethylrhodamine or fluorescent protein-labeled mitochondria.

Mitochondrial fission and fusion are highly dependent on cellular stress response signaling pathways (Lackner, 2013; Giacomello et al., 2020). Mitochondrial fission is regulated by the evolutionarily conserved dynamin-1-like protein (DRP1). In addition, the endoplasmic reticulum can wrap around mitochondria with actin and the DRP1 receptor, causing the release of mitochondrial fission factor, which promotes fission (Labbe et al., 2014). Mitochondrial fission can isolate damaged components, which can then be degraded by mitophagy (Ito and Di Polo, 2017). This mechanism is essential for the quality control of mitochondria (Das et al., 2020).

Mitophagy is the most compelling hypothesis for the selective autophagy of an entire mitochondrion and fragmented mitochondria. Autophagosomes recognize and endocytose dysfunctional mitochondria for their degradation. Mitophagy is induced by accumulating PTEN-induced putative kinase 1 (PINK1) and parkin (Narendra et al., 2008, 2010). PINK1 frequently translocates from the cytoplasm to the mitochondrial outer membrane. In the healthy mitochondria, PINK1 is constitutively repressed via its import into the inner mitochondrial membrane and is degraded by the rhomboid protease PARL (one of the mAAAs) (Yamano and Youle, 2013). When a mitochondrion becomes damaged, the import of PINK1 is prevented, and it therefore accumulates on the outer mitochondrial membrane (Meissner et al., 2015).

PINK1 on the outer mitochondrial membrane recruits parkin from the cytosol to ubiquitinate the impaired mitochondria and induces mitophagy (Eiyama and Okamoto, 2015). Optineurin is a parkin-mediated mitophagy receptor, and its recruitment to damaged mitochondria is an important downstream signal of parkin-mediated mitophagy (Lazarou et al., 2015). PINK1 accumulation is determined by severe mitochondrial depolarization. Therefore, when fission generates mitochondrial fragments, all of them are depolarized. The damaged fragments cannot restore their membrane potential and undergo mitophagy, whereas any healthy mitochondria maintain oxidative phosphorylation, restore their membrane potential, and undergo mitochondrial fusion to avoid mitophagy (Cho et al., 2019). Thus, this mechanism could be a novel model for mitochondrial quality surveillance.

Mitochondrial fusion consists of outer membrane fusion and inner membrane fusion. Membrane-anchored dynamin family members Mfn1 and Mfn2 mediate the fusion between mitochondrial outer membranes, whereas a single dynamin family member, optic atrophy 1 (OPA1), mediates fusion between mitochondrial inner membranes (Youle and van der Bliek, 2012). Enhancing mitochondrial fusion in glaucoma patients might ameliorate sub-clinical mitochondria damage by promoting the fusion with healthy mitochondria. Fused mitochondria are more capable of supplying ATP and are resistant to environmental stressors (Hoppins, 2014). Williams et al. (2012) demonstrated that OPA1 maintains the synaptic architecture and RGC connectivity. Further, OPA1 upregulation restores dysfunctional mitochondrial morphology and protects neurons against excitotoxic injury (Jahani-Asl et al., 2011).

In contrast, OPA1 deficiency leads to mitochondrial fragmentation, respiratory impairment, and calcium disturbances (Kushnareva et al., 2013; Sun et al., 2020). However, damaged mitochondria contaminate other mitochondria if they fuse with the mitochondrial network excessively before they are eliminated by autophagy (Youle and van der Bliek, 2012). Some scholars refer to these processes as the “dance between fusion and fission.”

### Defect of Mitochondrial Biogenesis, Motility, Dynamics, and Mitophagy in Glaucoma

Numerous studies have demonstrated mitochondrial abnormalities occurring in the neurodegeneration accompanying glaucoma in patients and animal ocular hypertension (OHT) models (Ju et al., 2008; Williams et al., 2017; Hass and Barnstable, 2019; Tribble et al., 2019, 2021). Previous studies have demonstrated that mitochondrial dysfunction is an early driver of neuronal dysfunction preceding clinically observable neurodegeneration (Williams et al., 2017).

Many studies have demonstrated that inhibiting neuronal PGC-1α activity impairs mitogenesis, thereby promoting neurodegeneration, as in Alzheimer’s disease and Parkinson’s disease (Pirooznia et al., 2020; Singulani et al., 2020). *In vitro* and *in vivo* experiments proved that enhanced AMPK/PGC-1α signaling pathway activity and PGC-1α expression protect RGCs in the RGC-5 cell line, rat primary RGCs, and rat chronic ocular hypertension models (Chen S. et al., 2013; Zhang et al., 2018; Uchida et al., 2019). A recent study showed that zinc is essential for PGC-1α transcription and increases antioxidant stress in human primary endometrial stromal cells (Lu et al., 2020). However, more research is needed to elucidate the role of zinc in PGC-1α signaling in neurons and RGCs.

The detection of real-time mitochondrial motility in human RGCs is limited owing to technical challenges. The explant model of the mouse eye and optic nerve enables the image analysis of the living optic nerve head, showing that the percentage of mitochondria in motion significantly decreases with an acute and chronic IOP elevation (Kimball et al., 2017, 2018). Nicotinamide, which protects the RGCs of DBA/2J mice, increases mitochondrial size and motility in primary RGC cultures (Tribble et al., 2021).

Mitochondrial defects drive many degenerative retinal diseases, and mitochondrial transplant restores function to RGCs in the retina with defective mitochondria (Jiang et al., 2019; Ferrington et al., 2020). However, the long-term consequences of manipulating the balance of mitochondrial dynamics to protect RGCs are unknown, which is a source of controversy when designing glaucoma treatments. Some studies indicate that increasing fission and mitophagy to flush out unhealthy mitochondria protects RGCs from glaucoma. Similarly, mitochondrial uncoupling protein 2 (UCP2) knock-out promotes mitophagy and decreases the death of RGCs in a chronic OHT mouse model (Hass and Barnstable, 2019). This assertion is easy to accept because autophagy is beneficial to longevity in most cases.

Dai et al. (2018) proposed that in early hypertensive rats, mitophagy is increased to compensate for the change in pressure, but the damage to RGCs progresses as mitophagy is impaired owing to lysosome dysfunction. Interestingly, parkin overexpression downregulates mitophagy in the first 3 days following IOP elevation and promotes mitophagy 2 weeks following IOP elevation, reducing RGC death in chronic hypertensive glaucoma rats (Dai et al., 2018). Parkin overexpression affects mitophagy via two different mechanisms in early and later stages of IOP perturbations, suggesting that manipulating mitophagy might be harmful to some patients. Mitophagic hyperactivity can result in an inadequate ATP supply and eventually trigger neuronal cell death, which has attracted increasing attention (Doxaki and Palikaras, 2020).

In humans with glaucoma and mouse glaucomatous models (including DBA/2J mice), the mitochondria in the soma, dendrites, and axon of RGCs are smaller, more rounded, and more fragmented than those in healthy humans or mice, suggesting a defect in mitochondrial fusion (Ju et al., 2008; Coughlin et al., 2015; Kim et al., 2015; Tribble et al., 2019). Some studies consider that the high fission and mitophagy in glaucomatous RGCs result from damage instead of successful adaptation, recommending caution when attempting therapeutic manipulations of these processes. This view might result from the phenomenon that cristae of fragmented mitochondria are structurally disrupted and less capable of producing ATP compared with whole mitochondria. For example, decreased fission mediated by DRP1 inhibition or increased fusion mediated by OPA1 overexpression rescues RGCs and their axons by preserving mitochondrial integrity (Park et al., 2011; Kim et al., 2015; Hu et al., 2018).

Under the use of different animal models, the complexity of spatio-temporal regulation, and the lack of standard mitophagy flux assay, there are multifaceted results reported on the role of mitochondrial dynamics and mitophagy in insulted RGCs, with mitochondrial fission and mitophagy either protecting or promoting cell death. Accordingly, the molecular mechanism of hyperactive mitophagy-induced loss of RGCs remains unclear and requires further research. Zhou et al. (2019) showed that dysregulated mitophagy is toxic to the body when mitochondrial permeability increases. In acute IOP elevation models, inhibiting the opening of the mitochondrial permeability transition pore (mPTP) reduces mitochondrial permeability and promotes RGC survival (Kim et al., 2014). In addition, excess iron and zinc have been shown to be the triggers for mPTP opening (Jiang et al., 2001; Rauen et al., 2004; Sripetchwandee et al., 2014). These findings might help us to better understand the role of mitophagy in glaucoma.

It is worth noting that the state of RGCs likely differs depending on the status of glaucoma. For example, widespread mPTP opening occurs shortly after reperfusion, which results in detrimental mitophagic cell death (Ong et al., 2015). The use of chloroquine, which inhibits autophagic flux, rescues the early-phase I/R injury in cells. However, in the late phase of reperfusion, the mPTP closes in mitochondria to restore functionality (Ma et al., 2015). Hence, mitophagy plays a beneficial role by selectively degrading dysfunctional mitochondria and improving cellular homeostasis in the late phase of I/R injury. It is thus necessary to comprehensively consider the state of mitochondria and select appropriate protective measures for glaucoma.

So far, we have summarized (1) the critical functions of iron and zinc in mitochondria; (2) the kinetics of these metal ions in cells and mitochondria; (3) the relationship of iron, zinc, and RGCs; and (4) the relationship of mitochondria and glaucoma. Since studies directly focusing the roles of iron and zinc in mitochondria of glaucomatous RGCs are limited, the detailed impacts of iron and zinc on the mitochondria of RGCs in glaucoma is far from clear. In the following sections, we provide an overview of discoveries of how metal ions affect mitochondria in the RGCs and the other neurons, which can help to infer the potential molecular mechanisms involved in glaucoma.

#### Effects of Iron on Mitochondria

Iron is crucial for mitochondrial biogenesis, motility, and dynamics in RGCs. The use of iron chelators is protective to RGCs in OHT and optic nerve injury models (Thaler et al., 2010; Cui et al., 2020). Previous studies demonstrated that iron deficiency caused by treating cells with iron chelators decreases mitochondrial biogenesis, increases mitochondrial mobility, and inhibits anterograde movement, which impair dendritic outgrowth and synapse formation during neuronal development (Bastian et al., 2019; Upadhyay and Agarwal, 2020). Excess iron accumulation increases intracellular Ca^2+^ and activates calcineurin, inducing mitochondrial fragmentation by dephosphorylating DRP1 (Park et al., 2015). Use of an iron chelator leads to mitochondrial elongation by decreasing Fis1 expression, a mitochondrial fission modulator (Yoon et al., 2006). Both iron overload and deficiency result in mitochondrial dysfunction. A detailed treatment window for iron chelators must be explored.

**FIGURE 3:**
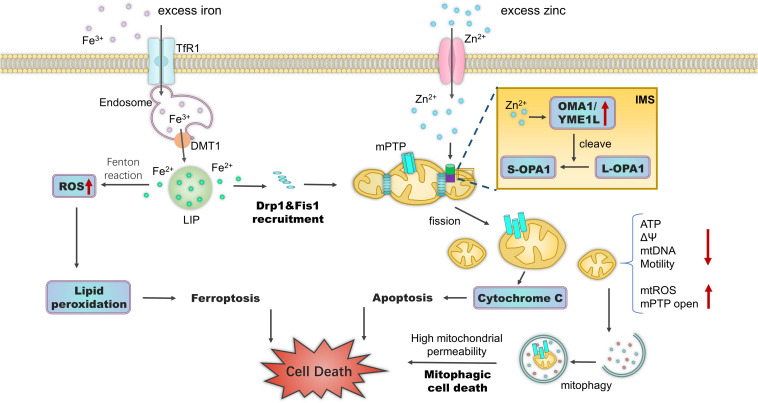
Effects of iron and zinc on mitochondria. Excess intracellular iron accumulation triggers mitochondrial fragmentation by increasing the expression of mitochondrial fission modulators Drp1 and Fis1. Fe^2+^ accumulation leads to the Fenton reaction and ferroptosis. Zn^2+^ accumulation and import into mitochondria via ion channels trigger mitochondrial depolarization, Drp1-dependent fission, and increased permeability. Under a range of stress stimuli, Zn^2+^ in the mitochondrial IMS activates zinc metallopeptidases OMA1 and YME1L to cleave long, fusion-active L-OPA1 to fusion-inactive S-OPA1 isoforms. This results in mitochondrial fragmentation and mitochondrial bioenergetic deficiency. During mPTP opening, fragmented mitochondria trigger mitophagic cell death. Zn^2+^ in the cytoplasm can also disrupt iron metabolism. Subsequent ferroptosis can occur via the over-accumulation of lipid ROS. Abbreviations: TfR1, transferrin receptor 1; DMT1, divalent metal transporter-1; LIP, labile iron pool; IMS, intermembrane space; ΔΨ, mitochondrial membrane potential; mtROS, mitochondrial reactive oxygen species.

In glaucoma, an increasing number of studies have offered evidence that ferroptosis contributes to RGC death due to the dysregulation of iron. Gao et al. (2019) proved that mitochondrial metabolism plays a crucial role in cysteine deprivation-induced ferroptosis. Ferroptosis induces a pathogenic mitochondrial morphology, including rupture of the outer mitochondrial membrane, reduction or disappearance of mitochondrial cristae, and changes in membrane potential (Vanden Berghe et al., 2014; Xie et al., 2016). Dexras1, essential for iron import in glutamate-NMDA neurotoxicity, promotes ferroptosis (Cheah et al., 2006; Peng et al., 2020). Deletion of Dexras1 in mice attenuates RGC death in NMDA/NO-mediated experimental glaucoma and optic neuritis (Chen Y. et al., 2013; Khan et al., 2019). FXN, a mitochondrial iron chaperone protein, is involved in ferroptosis by modulating iron homeostasis and mitochondrial function (Du et al., 2020). Suppressing FXN expression in RGCs results in enhanced mitochondrial fragmentation, undetectable cristae, impeded Fe-S cluster assembly, and enhanced ferroptosis. Further, FXN overexpression blocks erastin-induced ferroptosis (Du et al., 2020). Nevertheless, whether the protective role of FXN blocks experimental glaucoma owing to the suppression of ferroptosis remains unknown (Figure 3).

#### Effects of Zinc on Mitochondria

In glaucoma, Zn^2+^ import into mitochondria then leads to mitochondrial depolarization, fission, and the increase of permeability, which could be involved in RGC loss. Zn^2+^ accumulation, mPTP opening, and mitochondrial fission occur before neuronal apoptosis (Martin et al., 2011). As mentioned above, DRP1 activates the ZIP1-MCU complex, which imports Zn^2+^ into mitochondria, resulting in mitochondrial depolarization along with mitochondrial fission and Zn^2+^ accumulation in RGCs after optic nerve injury (Li et al., 2017). Recently, Ji et al. (2020) reported that using MCU knock-out or pharmacologic blockers significantly reduced mitochondrial Zn^2+^ accumulation, and attenuated Zn^2+^-triggered mitochondrial dysfunction and cortical neuron cell death. Using structured illumination microscopy and a new single Zn^2+^ fluorescent probe, Fang et al. (2021) revealed that CCCP-induced mitophagy in living HeLa cells was associated with mobile Zn^2+^ enhancement. It would be interesting to further explore whether mitophagy is deleterious under this probable mechanism in the early state of glaucoma.

There are studies provide links between zinc-induced ferroptosis and glaucomatous injury. Intracellular Zn^2+^ accumulation perturbs iron homeostasis and induces ferroptosis (Palmer et al., 2019). Chen et al. (2021) used genome-wide RNA interference screens to show that the zinc transporters ZIP7 and ZnT8, which control zinc movement between mitochondria and different cell compartments are essential for ferroptosis. The underlying mechanism was found to be related to mitochondrial ROS that activate AMPK-ULK1 signaling, triggering ferritinophagy (Qin et al., 2021). Ferritinophagy increases intracellular iron levels and subsequently results in oxidative injury via the Fenton reaction.

Zinc metallopeptidases regulate the mitochondrial dynamics of RGCs in glaucoma. The zinc metallopeptidases YME1L and OMA1 regulate the balance between long (L-OPA1) and short (S-OPA1) OPA1 protein forms through alternative splicing and proteolytic processing (Ehses et al., 2009). The generation of membrane-anchored L-OPA1 depends on zinc metallopeptidase MPP activity, which cleaves the OPA1 mitochondrial targeting sequence (Ehses et al., 2009). OMA1 cooperates with YME1L and converts L-OPA1 into the soluble S-OPA1 at the S1 and S2 sites, respectively (MacVicar and Langer, 2016). L-OPA1 induces mitochondrial fusion, whereas S-OPA1 does not. Moderately active OMA1 and YME1L maintain a steady-state balance between fusion and fission. Stress insults or metabolic cues can activate OMA1 or YME1L in RGCs (Rodriguez-Graciani et al., 2020). During aging and in diseases, increased OPA1 processing limits the content of fusion-active L-OPA1 and triggers mitochondrial fragmentation (Baburamani et al., 2015). OPA1 mutation is a cause of primary open-angle glaucoma (Huang et al., 2014). As discussed above, the upregulation of OPA1 protects RGCs in glaucoma (Hu et al., 2018). Epigallocatechin gallate (EGCG) supplementation was also shown to play a neuroprotective role on RGCs *in vitro* and in mouse models of retinal I/R and chronic glaucoma (Zhang et al., 2008; Shen et al., 2015). Indeed, EGCG directly decreased OMA1 activity by inhibiting the self-cleavage of OMA1, attenuating L-OPA1 cleavage, and maintaining mitochondrial function (Nan et al., 2019). However, it is unknown whether the increase in Zn^2+^ levels during the course of optic nerve injury can directly activate these zinc metallopeptidases. The zinc chelator TPEN significantly inhibits OMA1 activity in L-OPA1 cleaving, suggesting that zinc is necessary for enzymatic activity (Tobacyk et al., 2019). The association between OPA1 and mitochondrial zinc metallopeptidases can be another potential research focus with respect to providing insight into the roles of zinc and the mitochondria of RGCs in glaucoma (Figure 3).

## Challenges and Future Directions

Collectively, the findings summarized in this review indicate that metal homeostasis, whether involving metalloproteins or metal ions, is deterministic for the fate of RGCs. The overload of iron and zinc ions leads to the loss of RGCs, which can be alleviated by chelator treatment. In addition to metal ion levels, the forms and distribution of iron and zinc within cells are crucial for the normal function of RGCs, such as FXN and FtMt, which tune mitochondrial iron. Accumulating evidence indicates that mitochondrial abnormalities caused by iron or zinc accumulation are the underlying mechanism of glaucomatous injury. Including mitochondrial biogenesis and fusion deficiency, along with fission and mitophagy increases, among other processes, might participate in the loss process of RGCs.

However, there remains much to discover about the roles of iron and zinc in RGCs. Several questions still need to be solved, such as obtaining direct evidence of changes in iron and zinc levels in glaucomatous RGCs, the source of increased metal ions, where the ions go, and how they impact mitochondria. Importantly, for development of an effective glaucoma treatment, it is also important to determine the specificity of metal chelators, whether low metal concentrations would impair the synthesis and function of metalloproteins in the short or long term, and whether the surviving RGCs are still functional, which will necessitate further direct and detailed research. Moreover, how these essential metal ions, not only iron and zinc, interfere with each other in different cellular circumstances (for example, calcium assists ferroportin exporting iron) remains a challenge.

The gradual loss of RGCs in glaucoma, most of which is the result of apoptosis, is a chronic progressive process. Some patients still suffer from progressive loss of RGCs when using medication or surgery to maintain the IOP within acceptable limits. That is, RGCs undergo a long period of chronic stress before apoptosis occurs. Because mitochondria determine cell metabolism and fate in neurodegenerative diseases, the mitochondria of RGCs can play a vital role in this period. Specifying how metal ions influence the mitochondria might provide much needed insight into the bottleneck of glaucoma.

## Conclusion

This is an exciting time for research in glaucoma mitochondrial biology, with the emergence of several intriguing findings regarding mitochondrial biogenesis, dynamics, and quality control. RGCs are extremely dependent on mitochondria, and the function of mitochondria in glaucoma patients is of particular interest. Moreover, an increasing number of new studies has shown that both metalloproteins and metal ions participate in mitochondrial homeostasis and in the pathogenesis of glaucoma. It is important to investigate the roles of metals such as iron and zinc in the mitochondria of glaucomatous RGCs, despite many open questions remaining about the fundamental mechanisms underlying these processes. By utilizing advancements in single-cell and subcellular visualization technologies, it is expected that the mechanisms by which metals affect the mitochondria of RGCs in the development of glaucoma will be uncovered.

## Author Contributions

All authors, wrote and edited the manuscript, contributed to the article, and approved the submitted version.

## Conflict of Interest

The authors declare that the research was conducted in the absence of any commercial or financial relationships that could be construed as a potential conflict of interest.

## Publisher’s Note

All claims expressed in this article are solely those of the authors and do not necessarily represent those of their affiliated organizations, or those of the publisher, the editors and the reviewers. Any product that may be evaluated in this article, or claim that may be made by its manufacturer, is not guaranteed or endorsed by the publisher.
